# Supplementary material for: Bone Marrow-Derived Cells Contribute to the Maintenance of Thymic Stroma including TECs
Source: J Immunol Res. 2022 Apr 29;2022:6061746. doi: 10.1155/2022/6061746 (PMC9076333; doi:10.1155/2022/6061746)
Supplement: Supplementary Materials — Supplementary Figure 1: gating strategy for FACS analysis of the lineage-depleted bone marrow- and thymus-derived CD45+ EpCAM+ cell populations. (A) Gating strategy for live cells in bone marrow analysis: SSC A vs. FSC A; (B) gating for doublet discrimination of live cells: SSC W vs. SSC H; (C) gating strategy for doublet discrimination of live cells: FSC W vs. FSC H; (D) gating for lineage-negative single cells using a cocktail of lineage antibodies including CD11b, CD11c, and CD19; (E) the FACS representation of the lineage-depleted cells derived from bone marrow gated for CD45 and EpCAM; (F) the FACS representation of the lineage-depleted cells derived from bone-marrow gated stained with CD45 together with the EpCAM using isotype control used to set the gate for EpCAM staining; (G) the FACS representation of the lineage-depleted cells derived from the bone marrow of nude mice gated for CD45 and EpCAM; (H) gating strategy for live cells in the analysis of dissociated thymus: SSC A vs. FSC A; (I) gating for doublet discrimination of live cell: SSC W vs. SSC H; (J) gating for doublet discrimination of live cell: FSC W vs. FSC H; (K) gating for lineage-negative single cells using a cocktail of lineage antibodies including CD11b, CD11c, and CD19; (L) the FACS representation of the lineage-depleted cells derived from dissociated thymus gated for CD45 and EpCAM; (M) the FACS representation of the lineage-depleted cells derived from dissociated thymus gated for CD45 and EpCAM using the isotype control for EpCAM staining to allow proper gating of EpCAM expression. Supplementary Figure 2: FACS representation of the sort purity of bone marrow- and thymus-derived CD45+ EpCAM+ cells and the other control cell populations used for RNA isolation. (A) The FACS representation of the gating strategy used for sorting the thymus-derived CD45+ EpCAM+, CD45+ EpCAM-, and CD45- EpCAM+ subsets; (B-E) the FACS representation showing the purity of the live-gated (B) and thymus-derived CD45 [file 6061746.f1.docx]

**Supplementary Figures:**

Supplementary Figure 1:


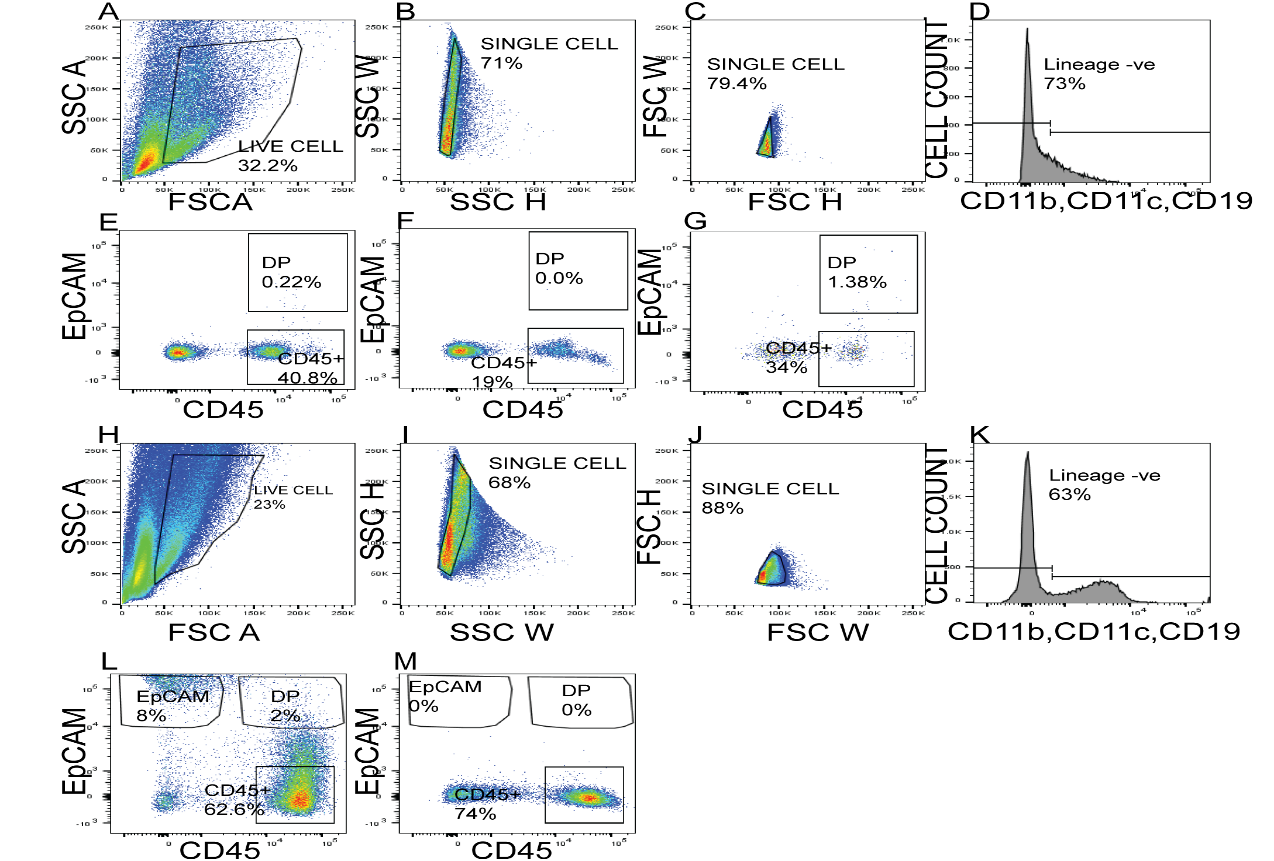


**Supplementary Figure 1**. Gating strategy for FACs analysis of the lineage depleted bone-marrow and thymus-derived CD45+EpCAM+ cell populations. A) Gating strategy for live cells in bone marrow analysis: SSC A vs FSC A. B) Gating for doublet discrimination of live cells: SSC W vs SSC H; C) Gating strategy for doublet discrimination of live cells: FSC W vs FSC H; D) Gating for lineage negative single cells using a cocktail of lineage antibodies including CD11b, CD11c, CD19; E) The FACS representation of the lineage depleted cells derived from bone-marrow gated for CD45 and EpCAM. F) The FACS representation of the lineage depleted cells derived from bone-marrow gated stained with CD45 together with the EpCAM using isotype control used to set the gate for EpCAM staining. G) The FACS representation of the lineage depleted cells derived from the bone-marrow of nude mice gated for CD45 and EpCAM. H) Gating strategy for live cells in the analysis of dissociated thymus: SSC A vs FSC A. I) Gating for doublet discrimination of live cell: SSC W vs SSC H; J) Gating for doublet discrimination of live cell: FSC W vs FSC H; K) Gating for lineage negative single cells using a cocktail of lineage antibodies including CD11b, CD11c and CD19; L) The FACS representation of the lineage depleted cells derived from dissociated thymus gated for CD45 and EpCAM. M) The FACS representation of the lineage depleted cells derived from dissociated thymus gated for CD45 and EpCAM using the isotype control for EpCAM staining to allow proper gating of EpCAM expression.

Supplementary Figure 2:


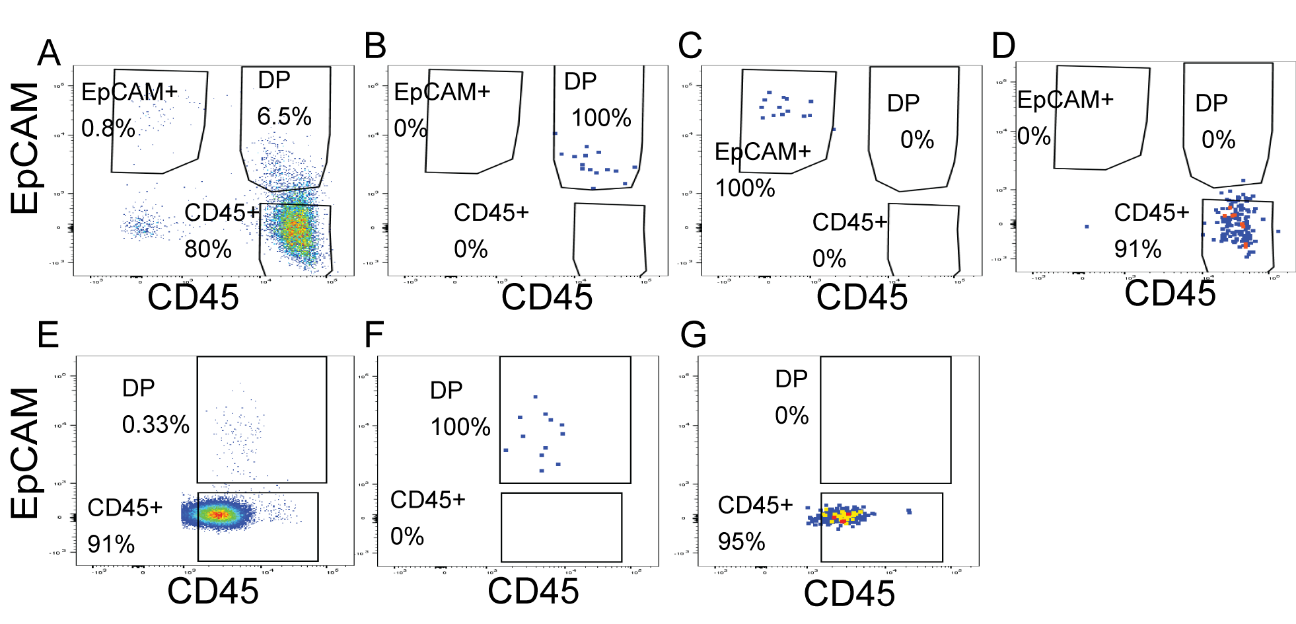


**Supplementary Figure 2.** FACS representation of the sort purity of Bone-marrow and thymus-derived CD45+EpCAM+ cells and the other control cell populations used for RNA isolation. A) The FACS representation of the gating strategy used for sorting the thymus-derived CD45+EpCAM+, CD45+EpCAM- and CD45-EpCAM+ subsets. B-E) The FACS representation showing the purity of the live gated (B) and thymus derived CD45+EpCAM+ (C) CD45-EpCAM+ (D) CD45+EpCAM- (E) subsets; F) The FACS representation of the gating strategy used for sorting the BM-derived CD45+EpCAM+ and CD45+EpCAM- subsets; G-I) The FACS representation showing the purity of the live gated (G) and BM-derived CD45+EpCAM+ (H) CD45+EpCAM- (I) subsets.

Supplementary Figure 3:


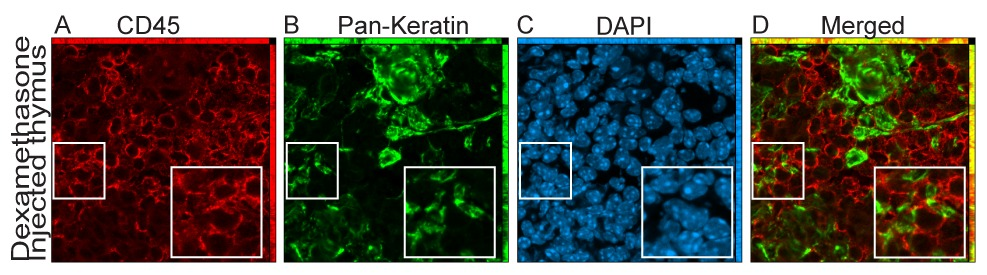


**Supplementary Figure 3.** Presence of a CD45+Pan-keratin+ population in adult thymic sections. Dexamethasone treated murine thymuses were sectioned and stained for CD45 and Pan-Cytokeratin. Panel A showing a Maximum Intensity projection of CD45 staining; Panel B showing Maximum Intensity projection of Pan-Keratin staining; Panel C showing Maximum Intensity projection of DAPI staining. Panel D showing Maximum Intensity projection of merged CD45 and EpCAM staining. CD45 and EpCAM expressing cell of interest is enlarged in the insets to show co-expression of both proteins and represent the cell shown in the white boxes.

Supplementary Figure 4:


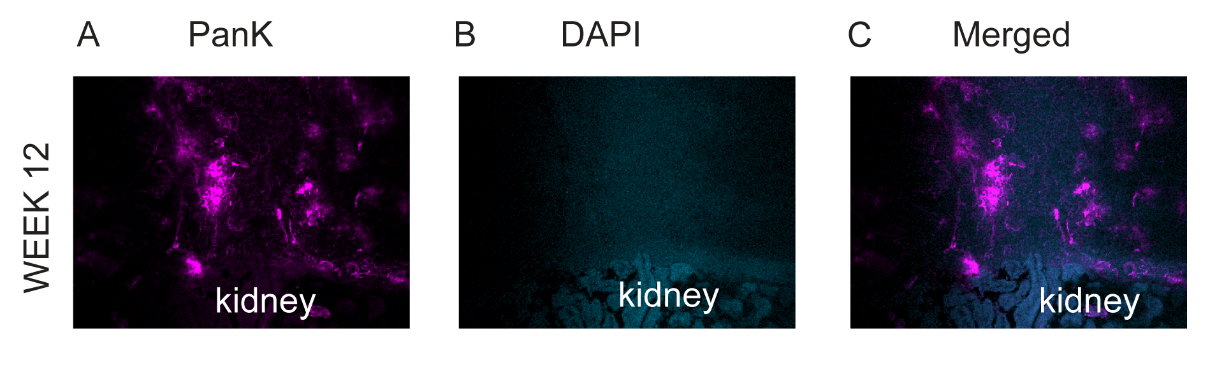


**Supplementary Figure** 4. A-C shows week 12 images of the transplanted fetal thymus. In this figure, the kidney is visible in the lower part of each image. This figure clearly shows a properly developed transplanted thymus at 12^th^ week.
